# Professional practices, collective dynamics, and psychosocial aspects of sailors on a French warship

**DOI:** 10.3389/fpsyg.2026.1930962

**Published:** 2026-10-29

**Authors:** Audrey Gohin, Léo Facca

**Affiliations:** Arts et Métiers Institute of Technology, École Navale, IRENAV EA3634, Brest, France

**Keywords:** collective dynamics, naval work, peer role, professional genre, real activity

## Abstract

**Background:**

Warship crews live and work for extended periods in confined spaces, separated from their families and exposed to high operational demands. While psychosocial risks in naval and maritime work are increasingly documented, little is known about how sailors collectively organize, regulate, and adapt their daily activity aboard combat vessels. To our knowledge, no psychological study has documented collective activity *in situ* aboard a French warship.

**Objective:**

Document sailors’ professional practices, collective dynamics, and psychosocial aspects *in situ* during an eight-day embarkation aboard a French multi-mission frigate.

**Method:**

This is a descriptive and qualitative study, of the experience report type, carried out in March 2026 during an eight-day embarkation aboard a French Navy European Multi-Mission Frigate taking part in a high-intensity exercise in the Atlantic. Data were collected through unstructured participant observation, from an observer-as-participant position. Inductive analysis drew on grounded theory and work psychology literature.

**Results:**

Three mechanisms emerged. First, rank-based messes provided spaces for peer interaction, informal regulation, and the expression of tensions. Second, sailors used situational leeway to adapt their activity to operational constraints and develop practical solutions. Third, interactions between experienced and less experienced sailors supported the transmission of knowledge, practices, and norms, contributing to collective memory and professional continuity.

**Conclusion:**

These findings illustrate how professional practices, collective dynamics, and psychosocial aspects are intertwined in sailors’ daily activity aboard a French warship. Informal collective spaces, opportunities to adapt work activity, and the transmission of the professional genre between experienced sailors and newcomers support professional activity.

## 1 Introduction

In March 2026, a work psychology researcher conducted an eight-day field immersion aboard a French Navy European Multi-Mission Frigate (FREMM), as part of a broader research project on the integration of emerging technologies into naval work environments. However, it also provided an opportunity to examine the everyday social and collective dimensions of naval work. The adoption of such technologies cannot be understood solely in terms of technical or cognitive factors. It is also shaped by the collective dynamics and social conditions within which sailors work, coordinate their activities, and negotiate the demands of their environment.

Research on military collectives has traditionally focused on group cohesion, as a driver of performance under pressure (Bartone et al., 2002; Siebold, 2007). More recent work has examined the stressors experienced by sailors and the importance of social resources in counteracting their effects (Hurtado et al., 2025; Schmied et al., 2024). However, few studies have examined the everyday practices through which collective activity is organized aboard operational vessels, where crews live and work together for extended periods, away from their families and friends. Sailors assigned to sea-going units are absent an average of 4 months per year, and some units experience even longer deployments (Assemblée nationale, 2019). How do sailors manage collective work under such conditions? How do they handle the tensions and conflicts that arise? Despite their relevance to understanding military work, these processes remain underexplored.

Work psychology offers a relevant theoretical and methodological framework for examining these dynamics. Concepts such as real activity and professional genre (Clot, 2010; Lhuilier, 2007) draw attention to the distinction between prescribed work and the activity that workers actually develop in practice. This perspective emphasizes the collective processes through which professionals transmit and transform occupational knowledge, coordinate their actions, negotiate ways of working, and develop situated responses to the problems encountered in real activity. Such approaches have been applied across a range of occupational settings, including healthcare, industry, and public services (Clot, 2008). To our knowledge, however, they have never been mobilized to examine collective activity aboard combat vessels.

International studies on sailors’ health highlight the ambivalent role of onboard relationships, which are experienced both as a resource and as a source of tension. Among naval personnel, social tensions on board contribute to psychological strain; conversely, peer support emerges as a resource during deployment for dealing with these tensions (Hazel et al., 2025; Hurtado et al., 2025). Among merchant seafarers, resources such as good working relationships, fair workload distribution, and social support are associated with lower levels of anxiety and depression. By contrast, exposure to workplace violence increases this risk (Thapa et al., 2026). More broadly, onboard living and working conditions emerge as the strongest predictor of psychological well-being (Jiang et al., 2026). In France, available research has focused mainly on sailors’ health. In the French Navy, Ferraty (2023) described the difficulties of adapting to life on board, related to restricted living space, confinement, prolonged separation, and possible exposure to danger. Le Claire et al. (2026) found that 54.1% of the 405 sailors surveyed, deployed aboard five surface ships, had taken at least one medication without a prescription during their mission. This finding suggests reliance on self-medication to cope with the constraints of deployment. Among civilian seafarers, studies have documented exposure to verbal aggression, sexual harassment, and potentially traumatic events (Sanz-Trepiana et al., 2024). Others have identified work strain, sleep constraints, isolation, and family separation among merchant Navy officer cadets (Coadic et al., 2024).

Taken together, these studies have improved knowledge of the stressors that sailors face and of the importance of resources, such as social support, in coping with them. Overall, they shed light on the psychosocial working conditions of sailors in both merchant and naval settings (Hurtado et al., 2025; Schmied et al., 2024; Thapa et al., 2026). However, they have mostly addressed these issues through self-reported experiences, questionnaires, health indicators, individual adaptation capacities (Van Wijk and Martin, 2021), or specific psychosocial factors. Even the available qualitative work relies on sailors’ retrospective accounts collected in focus groups (Schmied et al., 2024), rather than on direct observation of how collective activity is organized, regulated, and adapted *in situ* aboard an operational warship. To our knowledge, no previous study has examined, through direct observation of everyday activity aboard a French Navy warship, how professional practices, collective dynamics, and psychosocial processes are articulated. The collective is not only a potential source of social support; it is also central to how naval work is organized, regulated, and adapted in practice. What remains less understood is not simply whether sailors experience these constraints, but how they collectively organize, regulate, and adapt their everyday activity in response to them.

The present brief report addresses this gap through an observational study informed by a work psychology and psychosocial perspective. Drawing on field observations collected during an eight-day embarkation aboard a French Navy European multi-mission frigate (FREMM), the study examines selected aspects of sailors’ everyday collective activity, with particular attention to how work is organized, negotiated, and adapted in practice. Rather than providing an exhaustive account of life aboard the vessel, it uses selected observations to illustrate how collective activity develops at the intersection of formal organizational prescriptions, the ways in which sailors appropriate rules and professional norms and situated interactions among crew members. The objective of this study is therefore to document sailors’ professional practices, collective dynamics, and psychosocial aspects *in situ* during an eight-day embarkation.

## 2 Material and method

### 2.1 Study design and context of the research

This is a descriptive and qualitative study, of the experience report type, based on an eight-day field immersion conducted in March 2026 during an exercise in the Atlantic Ocean. It draws on unstructured participant observations conducted by a work psychology researcher aboard a FREMM specializing in anti-submarine warfare. The FREMM, on which the researcher embarked, is organized around several functional areas, including the bridge for navigation and maneuvering, the operations center for combat operations, the hangar and flight deck, the engine rooms, the medical area, the information technology and digital systems area, the personnel management and logistics area, and the ship’s safety command post. In addition to these operational areas, the vessel includes spaces devoted to meals and collective gatherings, relaxation, rest, and maintenance-related activities. During working hours or watch periods, sailors occupy posts associated with their functional responsibilities; in more private or semi-private spaces, such as lounges and berthing areas, they tend to regroup with peers or colleagues of similar rank.

The immersion took place during a high-intensity exercise involving several French Navy forces, designed to refine sailors’ detection and artillery practices. Although the content of the exercise cannot be described in detail for confidentiality and national security considerations, it can be noted that it also included mandatory onboard safety drills designed to detect and address potential incidents, such as fire, leakage, flooding, or toxic contamination.

### 2.2 Participants and access to the vessel

Access to the vessel was obtained through a formal embarkation request. The researcher, affiliated with the French naval academy, submitted a request to the Director of Officers, who facilitated contact with available vessels. The commanding officer of a FREMM agreed to host the researcher for an eight-day embarkation. The purpose of this embarkation was to observe sailors’ professional practices. Prior to the embarkation, the commanding officer informed the crew that a researcher would be onboard and that they were free to speak with her or not. All crew members were thus aware that the study was taking place.

### 2.3 Researcher’s position and conduct of the immersion

During the embarkation, the researcher conducted unstructured participant observations. Her role was primarily observational, although she also engaged in informal interactions with crew members, corresponding to an observer-as-participant position (Gold, 1958). She observed sailors’ professional practices during exercises and, when possible, asked them questions (only with the sailors who had spontaneously spoken to her beforehand) either during the activity or afterward about the behaviors, decisions, and adjustments they had made or were making. She observed and/or interacted informally with a large proportion of the crew, including personnel across ranks and status groups, such as officers, non-commissioned officers, senior non-commissioned officers, and enlisted sailors. The researcher carried out all these activities in order to better understand the sailors’ work environment but also to engage in interactions based, as far as possible, on mutual trust. Her role was therefore to become acculturated to this environment, to respect its rules and customs, in order to be accepted in return by the crew:

Whatever the researcher’s status on board, they are now ‘embarked’ in this unusual field, which nevertheless makes them, for a time, a participant in the place, its inhabitants and their destinations, even the most unexpected. They are first and foremost a civilian passenger who must comply with the daily constraints of military life^1^. (Trompette et al., 1999, p.10).

This position implied a continuous presence in work and living spaces, without prior selection of observed situations. The researcher thus shared the crew’s daily life, took part in meals, attended exercises and debriefings, and engaged in informal exchanges with sailors who came to her spontaneously. Observations were conducted in all functional areas of the FREMM to which she had access, for example the bridge, the safety command post, and the engine rooms. Only the operations center and the information technology and digital systems area command post were not accessible to the researcher, as these areas were restricted to authorized personnel.

### 2.4 Data collection

To track her observations, the researcher kept a field logbook. This tool enabled her to record what she perceived and heard about life on board over the eight-day immersion. Each evening, she revisited her daily notes, expanding them with reflexive comments, distancing herself from immediate impressions, and organizing them in a more systematic manner until theoretical saturation was reached. After the embarkation, she cross-referenced her field notes with the existing scientific literature. The resulting material covered a wide range of situations, including sailors’ activities at different posts, the general organization of the vessel, self-presentation strategies during debriefings, formal procedures, and everyday interactions on board. In terms of data analysis, the researcher adopted an inductive approach informed by *grounded theory* principles (Charmaz, 2014; Glaser and Strauss, 1999). The analytical categories presented in the findings were not defined *a priori*; rather, they progressively emerged from the field notes through constant comparison across situations observed over successive days, including exercises, debriefings, meals, and informal interactions. Among the themes that emerged from these observations, the organization of collective life on board, and its effects on sailors’ work collectives, appeared particularly salient for two reasons. First, this focus was consistent with the researcher’s disciplinary orientation in work psychology and her socioconstructivist grounding, which conceptualizes work as situated activity embedded in a broader social and organizational context. Second, it resonated with the commanding officer’s interest in better understanding collective dynamics within the warship. The researcher therefore agreed to provide him with oral feedback on this issue, summarizing what she had observed and analyzed regarding the crew’s collective functioning on board. This feedback included both interpretive observations and practical suggestions.

### 2.5 Ethical considerations

The study complied with the Code of Ethics for Psychologists and the Declaration of Helsinki. The entire crew was informed by the FREMM’s commanding officer, prior to embarkation, of the researcher’s presence and the purpose of the study. In accordance with ethical considerations, the researcher only observed and engaged in informal interactions with sailors who came to her spontaneously to speak. No formal interviews were conducted, including on the theme of the collective onboard. Anonymity was guaranteed, and no sensitive data were collected.

### 2.6 Scope of the study

Consistent with the inductive method (Blais and Martineau, 2006), this study does not seek statistical generalizability but offers a contextualized understanding of the observed dynamics. The findings presented in the following section draw on the researcher’s field logbook and on the scientific literature revisited after the embarkation. They do not claim to be exhaustive: they offer a situated reading of selected aspects of collective life onboard, based on observations conducted during the eight-day immersion.

## 3 Results

### 3.1 The rank-based messes as a space for peer support and tension regulation

Life at sea is characterized by constant proximity, a pronounced permeability between private and professional life, and separation from one’s shore-based social network, although this separation is no longer complete given the availability of digital communication tools. The crew therefore lives in a kind of social and spatial “bubble,” in which spaces and times devoted to formal work are closely intertwined with those dedicated to more informal interactions. In demanding work situations, family members and friends can ordinarily provide external resources for coping with occupational strain. At sea, however, these external resources are less readily available. Family and friends can help with emotional regulation when a conflict arises among sailors, but what about at sea, when those sources of support are far away? During an exercise conducted aboard the FREMM, the researcher observed tensions between crew members stemming from divergent views about the most appropriate strategy for carrying out the task. The researcher identified two spaces in which such tensions could be expressed and regulated. The first was formal: the post-exercise debriefing functioned as a structured feedback setting in which crew members could voice disagreements within a framework specifically designed for collective analysis. During these debriefings, contradictions were discussed collectively in order to make sense of unexpected events encountered during the exercise and to reconsider the activity with a view to improvement. The second space was more informal. In the evening, before meals, sailors gathered with peers of the same rank or hierarchical level for a moment of relaxation. These rank-based spaces, called “messes,” provided an opportunity to express emotions, release frustrations accumulated during the day in one’s functional role, and feel understood by others who shared similar constraints. Peers thus played an important role in listening, support, and the regulation of tensions. This organization into rank-based messes - a distinctive feature of large Navy vessels - and the peer role associated with them may therefore help transform tensions experienced at work into something that can be spoken about, shared, and ultimately worked through. Although the researcher did not directly observe such situations, the reverse process may also be considered: when tensions arise within the mess, among peers, formal work colleagues may in turn assume a regulatory function. This spatial and social differentiation may therefore be particularly important because it can partly substitute for the emotional and social support usually provided by family members when such support is less accessible at sea. On newer frigates, either recently delivered or still under construction, the organization of private spaces is expected to change, with some spaces being merged in favor of more functionally oriented layouts. A senior non-commissioned officer, questioned by the researcher in an informal discussion, expressed concern about this development. In his view, the merging of messes increases hierarchical proximity, as ranks become more mixed within the same private spaces, thereby reducing opportunities for free expression within rank-based collectives. For this sailor, the implications of this spatial reconfiguration were clear: without a dedicated “release valve” for expressing tensions, there may be a greater risk of animosity, unspoken resentment, and persistent conflict.

### 3.2 Providing a creative space and situational leeway

On board, other practices also contributed to creating spaces for creativity and social bonding, including humorous morning announcements, joke exchanges among colleagues, and video game sessions in the mess. These activities allowed crew members to relax and build interpersonal ties, but also to express themselves beyond formal procedures, produce shared cultural content, and play with the codes of everyday life on board. Like peer exchanges, these creative - or, more precisely, recreational - spaces appeared to function as resources for social bonding. For instance, the researcher heard, as did the rest of the crew, morning announcements from sailors on the bridge who used excerpts from well-known songs to present the day’s activities or to send a message to another sailor celebrating a birthday.

Creativity, however, was not limited to recreational forms of expression; it was also embedded in work activity itself. The researcher observed this practical creativity in sailors’ everyday work: “I do it this way; it’s easier,” “If I put it here, it will hold better,” or “Wait, normally we do it like this, but if you go this way, it will be faster and produce the same result.” Such ordinary adjustments help make work possible by facilitating action, arbitrating between constraints, and adapting prescribed procedures to situated demands. On the FREMM where the field immersion took place, the commanding officer trusted crew members and allowed them such leeway. Sailors were therefore able to use this flexibility to develop situated ways of working, imprinting a “signature” on their activity while collectively adapting to constraints and unexpected events.

### 3.3 The second role of peers: transmitting the “professional genre”

Peer sailors - either of the same rank as the newcomer or of the rank to which the newcomer aspires - who had already been at sea for several months or years transmitted skills, gestures, postures, and practical tips to less experienced crew members. For example, the researcher observed a watch supervisor on the bridge teaching a mnemonic strategy to a midshipman to help her remember key information to report to the navigating officer, her hierarchical superior. The spaces in which this transmission occurs are often those in which collective work enables rules to be adjusted and mutual assistance among professionals to emerge. The construction of sailors’ experience is therefore inseparable from collective dynamics that support the transmission of practical knowledge, gestures, and norms. It may thus be particularly relevant to rely on peers who are positioned to sustain this transmission and to facilitate the organizational socialization of newcomers to the Navy.

## 4 Discussion

The originality of this study lies in both its field setting and its methods of data collection and analysis. Regarding the field setting, researcher immersions aboard combat vessels remain rare. These 8 days spent aboard a FREMM during a high-intensity exercise made it possible to observe sailors’ real activity (Clot, 2010) as it unfolded, whereas existing studies rely mainly on questionnaires or retrospective accounts (Schmied et al., 2024; Thapa et al., 2026). As for the analysis, the concepts of professional genre (Clot, 2008; Lhuilier, 2007) and work collective (Clot and Caroly, 2004) had not, to our knowledge, previously been used to study collective life on board. Yet they make it possible to capture what sailors concretely do together, day to day, to keep their work going.

The work collective observed on board appeared to us to be relatively balanced. Peer relationships played a supportive role, and the commanding officer placed his trust in the crew by granting it situational leeway (Coutarel et al., 2013). In addition, experienced sailors actively passed on the professional genre to newcomers. Research on military cohesion has mainly approached it as a lever for performance under pressure (Bartone et al., 2002; Siebold, 2007). Our observations suggest that collective life on board cannot be approached solely from the perspective of performance. Research conducted among shipboard sailors points in the same direction, identifying socialization opportunities and peer support as important resources during deployment (Hurtado et al., 2025; Schmied et al., 2024). Our study adds to this work by showing how these resources are embedded in the everyday organization of collective activity. Three mechanisms emerge from our observations: the role of rank-based messes in regulating tensions among peers, the leeway that makes room for real activity, and the transmission of the professional genre from experienced sailors to newcomers. These mechanisms can be interpreted through the theoretical framework of work psychology.

The mess, as a compartmentalized space for relaxation and self-disclosure (Trompette et al., 1999), appears to function as a structural “release valve,” enabling tensions experienced at work to be verbalized, shared, and potentially worked through, thereby supporting recovery from work (Sonnentag and Fritz, 2007). Studies conducted among United States Navy sailors identify camaraderie, moments of sociability, and organized social events on board as among their main means of coping with stress at sea (Hurtado et al., 2025; Schmied et al., 2024). Recreational activities and the role of music also constitute important resources (Janssen et al., 2026). The morning announcements set to songs and the video game sessions observed aboard the FREMM follow the same logic. Beyond the recreational function of collective events, onboard living and working conditions emerge as the strongest predictor of sailors’ psychological well-being (Jiang et al., 2026). The authors relate these conditions to the layout of living spaces, interpersonal relationships, and relaxation areas. Our observations suggest that the organization of space matters: by separating work areas from relaxation areas, and messes by rank, it makes these moments of regulation possible. Research on military groups, for its part, emphasizes the role of strong cohesion in mitigating the effects of isolation (Cojocaru and Lungu, 2013; Podorozhnii and Ivanov, 2024; Siebold, 2007). However, the present study provides a more nuanced perspective by examining the dialectic between cohesion and conflict. Although tensions and conflicts may be avoided or suppressed because they are experienced as uncomfortable and have been associated with adverse outcomes for well-being, including depressive symptoms and burnout (De Dreu et al., 1999; Finch et al., 1999; Sözen and Başım, 2023), our observations suggest that such tensions may also be part of the collective’s functioning and contribute to its regulation by reshaping interpersonal relationships and group dynamics, provided that they do not become persistent or destructive (Coser, 1956). This distinction calls for a more dynamic and process-oriented understanding of collective life onboard, in which cohesion does not necessarily imply the absence of tensions or conflict. Rather, tensions may form part of the processes through which collective relations are negotiated and regulated. From this perspective, conflict should not be understood solely as a threat to be neutralized, but also as a potentially constructive process that, when expressed and regulated within dedicated spaces, may help clarify expectations, readjust roles, and ultimately support the collective’s capacity to work together under pressure.

The concept of “real activity” (Clot, 2010) and the situational leeway granted by the commanding officer (Coutarel et al., 2013) highlight the dialectical relationship between prescribed and real activity: prescribed activity shapes real activity, while real activity may, in turn, lead prescriptions to evolve. The observations collected aboard the FREMM illustrate this dialectic. The ordinary remarks of sailors such as “I do it this way; it’s easier,” “If I put it here, it will hold better” show that these adjustments are not mere deviations from the norm: they make work possible, arbitrate between competing constraints, and adapt procedures to the situated demands of action (Engeström, 2001). It is precisely in this gap between the prescribed and the real that the meaning of work develops. This perspective enables sailors to develop their “power to act” (Clot and Simonet, 2015), particularly when they benefit from situational leeway within the organization of work. The observations suggest that the commanding officer of the FREMM granted precisely the kind of leeway that allows sailors’ power to act to develop.

The development of the “power to act” facilitates the development of the “professional genre” (Clot, 2008). The transmission of the “professional genre” (Lhuilier, 2007) from experienced sailors to novices further sustains the collective memory of the trade and facilitates the organizational socialization of newcomers (Demazière et al., 2019). It allows them to avoid having to reinvent everything and to integrate into a collective already carrying implicit rules. Thapa et al. (2026) recommend developing peer mentoring, particularly during the first months on board; our observations show that it already exists informally. For example, observing a watch supervisor teach a midshipman a mnemonic strategy for remembering the information to be reported to the navigating officer provides a concrete illustration of this process. What is transmitted here is not merely a technique; it is also a way of relating to the profession - a way of carrying oneself, communicating, prioritizing, and coordinating with others. Such transmission presupposes spaces in which collective work enables professional rules and practices to be discussed and adjusted (Clot and Caroly, 2004). The mess, debriefings, and informal interactions that characterize life onboard constitute precisely such spaces. These observations are consistent with recent research on the psychosocial vulnerabilities of naval personnel (Coadic et al., 2024; Ferraty, 2023; Le Claire et al., 2026; Sanz-Trepiana et al., 2024). Rather than contradicting these findings, the present study places them within the everyday organization of collective activity. Rank-based messes, situational leeway, and the transmission of the professional genre appear to function as collective resources that may mitigate—though not eliminate—the psychosocial strains identified in this literature.

Despite these contributions, this study has several limitations. First, the researcher’s position as an observer-as-participant (Gold, 1958) may have introduced several sources of bias, including social desirability on the part of sailors during interactions with the researcher and potential observer effects related to the researcher not being subject to the same disciplinary rules as the crew. Second, restricted access to certain areas of the ship, such as the operations center and information technology and digital systems area command post, may have limited the scope of the observations, particularly regarding collective work in “high-intensity” situations. Third, the study did not include formal interviews or focus groups specifically addressing the work collective, limiting our ability to examine participants’ own interpretations of the observed dynamics. Nevertheless, the study retains heuristic value. Few studies in psychology have gained access to an operational warship, and this eight-day immersion provides rare empirical material on sailors’ collective activity. The application of French and international work psychology concepts (e.g., “professional genre,” “real activity,” and “work collective”) to the context of the French Navy also offers a relatively underexplored perspective, as research on naval personnel has predominantly relied on surveys or retrospective accounts (Hurtado et al., 2025; Schmied et al., 2024). Furthermore, the inductive and qualitative methodology enables a contextualized understanding of the observed dynamics (see Patton, 2015).

## 5 Conclusion

This study set out to document sailors’ professional practices, collective dynamics, and psychosocial aspects *in situ* during an eight-day embarkation aboard a French multi-mission frigate. Three main findings emerged. First, collective life onboard cannot be reduced to cohesion; rather, it relies on differentiated spaces and roles through which tensions can be expressed and regulated. Second, the organization of work allows for situated adjustments that contribute to making activity meaningful, provided that sufficient situational leeway is granted by the chain of command. Third, the transmission of practical knowledge and professional norms between experienced sailors and newcomers supports the continuity of the trade and the socialization of new crew members. Taken together, these findings suggest that the psychosocial dimension of naval work is not an adjunct to operational effectiveness but one of the conditions that sustain it.

These findings also point to several implications for the organization of work onboard. Rank-based messes may be considered not only as spaces for rest and social interaction, but also as resources for collective regulation and should therefore be taken into account when designing future vessels. This is consistent with recent recommendations to improve onboard living conditions and to increase opportunities for social activities at sea (Jiang et al., 2026; Schmied et al., 2024). Similarly, preserving opportunities for situational leeway may support the expression of real activity and enable crews to adapt their practices to the demands of specific situations. The role of experienced sailors, particularly senior non-commissioned officers, as carriers and transmitters of the professional genre could also be more explicitly acknowledged, both to support meaning at work and to facilitate the organizational socialization of newcomers, in line with calls to develop peer mentoring on board (Thapa et al., 2026). More broadly, these mechanisms may be relevant to the design of collective work in other confined and isolated environments.

These findings open several avenues for future research. Further observational studies combining *in situ* observation with interviews could examine whether similar mechanisms operate across different types of vessels and within other branches of the French armed forces. Longitudinal research could also examine how situational leeway, peer regulation, and the transmission of professional knowledge evolve over the course of a deployment or career. Finally, future research should investigate how emerging technologies, particularly artificial intelligence, interact with these collective mechanisms and potentially reshape the organization, regulation, and transmission of work in naval environments.

## Data Availability

The original contributions presented in the study are included in the article/supplementary material, further inquiries can be directed to the corresponding author.

## Footnotes

1 Free translation by the author of the article originally written in French.
